# Anaerobic Degradation of Naphthalene and Pyrene by Sulfate-Reducing Cultures Enriched from Former Manufactured Gas Plant Soil

**DOI:** 10.1007/s00248-022-02042-4

**Published:** 2022-05-24

**Authors:** Kartik Dhar, Logeshwaran Panneerselvan, Suresh R. Subashchandrabose, Kadiyala Venkateswarlu, Mallavarapu Megharaj

**Affiliations:** 1 Global Centre for Environmental Remediation (GCER), College of Engineering, Science and Environment, The University of Newcastle, ATC Building, University Drive, Callaghan, NSW 2308 Australia; 2 Cooperative Research Centre for Contamination Assessment and Remediation of the Environment (CRC CARE), The University of Newcastle, ATC Building, Callaghan, NSW 2308 Australia; 3 Formerly Department of Microbiology, Sri Krishnadevaraya University, Anantapuramu, 515003 India

**Keywords:** Anaerobic biodegradation, Naphthalene, Pyrene, Sulfate-reducing bacteria, *Desulfotomaculum* spp

## Abstract

Biodegradation of polycyclic aromatic hydrocarbons (PAHs) under completely anaerobic sulfate-reducing conditions is an energetically challenging process. To date, anaerobic degradations of only two-ringed naphthalene and three-ringed phenanthrene by sediment-free and enriched sulfate-reducing bacteria have been reported. In this study, sulfate-reducing enrichment cultures capable of degrading naphthalene and four-ringed PAH, pyrene, were enriched from a contaminated former gas plant site soil. Bacterial community composition analysis revealed that a naphthalene-degrading enrichment culture, MMNap, was dominated (84.90%) by a Gram-positive endospore-forming member of the genus *Desulfotomaculum* with minor contribution (8.60%) from a member of *Clostridium*. The pyrene-degrading enrichment, MMPyr, was dominated (97.40%) by a species of *Desulfotomaculum*. The sequences representing the *Desulfotomaculum* phylotypes shared 98.80% similarity to each other. After 150 days of incubation, MMNap degraded 195 µM naphthalene with simultaneous reduction of sulfate and accumulation of sulfide. Similarly, MMPyr degraded 114 µM pyrene during 180 days of incubation with nearly stochiometric sulfate consumption and sulfide accumulation. In both cases, the addition of sulfate reduction inhibitor, molybdate (20 mM), resulted in complete cessation of the substrate utilization and sulfate reduction that clearly indicated the major role of the sulfate-reducing *Desulfotomaculum* in biodegradation of the two PAHs. This study is the first report on anaerobic pyrene degradation by a matrix-free, strictly anaerobic, and sulfate-reducing enrichment culture.

## Introduction

Polycyclic aromatic hydrocarbons (PAHs) are a group of compounds composed of two or more fused benzene rings. These ubiquitous environmental pollutants originate during incomplete combustion of carbonaceous materials besides occurring naturally in crude oil, coal tar, creosote, and coal. Some PAHs are regarded as priority environmental pollutants due to their toxic, mutagenic, and carcinogenic properties [1, 2]. Despite the persistence conferred by their complex and rigid ring structure, high resonance energy, and limited bioavailability, PAHs are susceptible to microbial degradation under aerobic and anaerobic conditions following different alternative electron-accepting processes [3–10]. Anoxic environments such as subsurface soil, fresh and marine sediments, and sludge are important sinks of PAHs. However, aerobic microbial transformation of PAHs in such environments is limited due to the lack of oxygen that acts as the final electron acceptor in the respiration process. Anaerobic PAHs degradation following alternative electron accepting processes is challenging, particularly due to the recalcitrance of the contaminants, extremely slow growth of anaerobic microorganisms, and considerably low energy yield from the substrate mineralization processes. Despite all such odds, several studies demonstrated anaerobic biodegradation of two- to five-ringed PAHs under nitrate-reducing, iron-reducing, sulfate-reducing, and methanogenic conditions [4, 5].

Dissimilatory sulfate reduction is an essential process in anoxic environments where sulfate-reducing bacteria (SRB) and archaea participate in the biogeochemical cycling of redox active elements and play a crucial role in organic carbon decomposition [11, 12]. SRB predominate in anoxic environments where sulfate is abundant, for example, in marine sediments. They can also colonize in freshwater sediments that are generally low in sulfate (10–200 µM) [13]. The involvement of SRB in the biodegradation of naphthalene, 1-methylnaphthalene, and phenanthrene has been established in laboratory enrichments and pure cultures. Notable among the enrichments are *Deltaproteobacteria* dominant in naphthalene and 2-methylnaphthalene-degrading culture N47 [14], *Desulfobacteraceae* dominant in phenanthrene-degrading TRIP1 [15], and deltaproteobacterial phenanthrene-degrading culture obtained by Davidova et al. [16]. Only three naphthalene-degrading pure cultures, NaphS2, NaphS3, and NaphS6, all belonging to the *Desulfobacteraceae*, have been described so far [17, 18]. Recently, Zhang et al. [19] reported phenanthrene degradation under sulfate-reducing conditions by a pure culture of *Desulfotomaculum* sp. However, reports on ≥ four-ringed high molecular weight (HMW) PAHs degradation under sulfate-reducing conditions are scarce. The available reports described anaerobic biodegradation of HMW PAHs in sediment and sludge microcosms [20–24]. Anaerobic biodegradation of HMW, four-ringed pyrene by a matrix-free sulfate-reducing culture, has not been reported yet.

Most of the successful sulfate-reducing PAHs-degrading cultures have been enriched from hydrocarbon-contaminated anoxic freshwater or marine sediments [16, 18, 25]. Most frequently, the main players in PAHs degradation have been affiliated with Gram-negative SRB group belonging to the family *Desulfobacteraceae*. SRB are ubiquitous in soil; especially Gram-positive spore forming SRB can survive, find a niche in soil micropores for protecting themselves from direct exposure to oxygen, and even multiply in aerated topsoil [13, 26]. Therefore, long-term contaminated soils may harbor hydrocarbon degrading SRB. However, studies on anaerobic biodegradation by soil SRB are rare, probably due to the assumption that the bacteria are dormant in oxic environment. Nevertheless, Boopathy [27] demonstrated diesel fuel degradation in soil column under anaerobic sulfate-reducing conditions. Interestingly, Abu Laban et al. [28] enriched a benzene-degrading culture that was dominated by the Gram-positive sulfate-reducing *Pelotomaculum* using soil from a former coal gasification site. In this study, we enriched naphthalene- and pyrene-degrading bacteria under strictly anaerobic sulfate-reducing conditions from a long-term PAHs contaminated manufactured gas plant site soil. The PAHs-degrading sulfate-reducing bacterial communities in the soil-free enrichment cultures, obtained after repeated subcultures over a period of 2.5 years, were analyzed based on 16S rRNA sequencing. Moreover, the extents of naphthalene and pyrene utilization in conjunction with the substrate-dependent sulfate reduction activity have been established.

## Materials and Methods

### Soil Sample

The soil sample was collected from a former MGP site at 0–20 cm depth. The site, which was operated as gasworks during 1913–1985, is located in the city of Newcastle, New South Wales, Australia. No special precaution for protecting the sample from the air was taken during collection, transportation, and storage. The sample was stored in an amber glass jar at room temperature for 10 days before developing the enrichment cultures. The soil was ground and sieved (2 mm) inside a Don Whitely A35 anaerobic workstation under N_2_:CO_2_:H_2_ (80:10:10) environment. PAHs in the soil sample were extracted following our previous protocol [29] with the following modification. The dry residue after the solvent evaporation was reconstituted in 1.0 mL hexane, and the USEPA 16 PAHs were analyzed by gas chromatography-mass spectrometry (GC–MS). The GC–MS operating parameters are provided in the following section.

### Enrichment of Sulfate-Reducing Cultures with PAH-Degrading Abilities

The enrichment cultures were developed using 10 g soil and 90 mL reduced, and bicarbonate buffered mineral medium contained in 100 mL serum bottles. The reduced and bicarbonate buffered mineral medium was prepared according to the composition of Widdel [30] with some modifications. The medium contained, per liter of water, NaCl, 1.0 g; MgCl_2_·6H_2_O, 0.40 g; CaCl_2_·2H_2_O, 0.10 g; NH_4_Cl, 0.25 g; KH_2_PO_4_, 0.20 g; KCl, 0.50 g; Na_2_SO_4_, 4.26 g; 1.0 mL non-chelated trace element solution A, 1.0 mL trace element solution B, 1.0 mL vitamin mixture, 1.0 mL thiamine solution, 1.0 mL vitamin B_12_ solution, and 1.0 mL riboflavin solution. The medium was buffered with 30 mL of 1.0 M NaHCO_3_ solution and reduced with 5 mL of 0.20 M Na_2_S·9H_2_O solution. Resazurin at 5 mg L^–1^ final concentration was added as the anaerobic condition indicator. The pH of the medium was adjusted to 7.0–7.20 with HCl. Four milliliter stock solution of naphthalene (40 mM) or pyrene (20 mM) prepared in sterile 2,2,4,4,6,8,8-heptamethylnonane (HMN) was added as an overlay. The bottles were closed with butyl rubber stoppers and secured with aluminum crimp seals under an atmosphere of N_2_:CO_2_:H_2_ (80:10:10). In addition, controls of (a) sterile soil and (b) HMN without any dissolved PAHs were maintained. The bottles were incubated inside the anaerobic workstation at 25 °C in an inverted position to avoid direct contact between HMN layer and rubber stopper. Enrichments showing substantial sulfate reduction and sulfide accumulation relative to the control during incubation were further subcultured on fresh medium using 10% (v/v) inoculum. The naphthalene- and pyrene-degrading sulfate-reducing soil-free enrichment cultures were obtained after repeated transfers. Bacterial diversity profiling and biodegradation experiments were performed with sixth subculture of the enrichments.

### Evaluation of Sulfate-Reducing Enrichment

Lactate yeast extract (LYE) basal medium was prepared in 1.0 L Duran bottle according to the following composition (per liter of water): NaCl, 1.0 g; MgCl_2_·6H_2_O, 0.40 g; Na_2_SO_4_, 4.0 g; NH_4_Cl, 0.25 g; KCl, 0.20 g; yeast extract 1.0 g, and sodium DL-lactate, 2.25 g. The basal medium was sterilized by autoclaving at 121 °C for 5 min. Soon after autoclaving, the hot (≥ 50 °C) medium was purged with sterile N_2_ until cooling down to room temperature (~ 25 °C). The medium was sterilized again at 121 °C for 15 min after tightly capping the Duran bottle. The sterile medium was immediately transferred inside the anaerobic workstation and allowed for gas exchange for 48 h. The basal medium was supplemented with 10 mL of freshly prepared 5% (w/v) FeSO_4_·7H_2_O and 0.70 mL 1 M CaCl_2_·2H_2_O just before use. The pH was adjusted to 7.0–7.20. The LYE medium (10 mL) was distributed in Hungate tubes, inoculated with 0.50 mL enrichment culture under anaerobic conditions, and incubated at 25 °C. Viable inoculum with active sulfate-reducing bacteria was indicated by blackening of the medium due to FeS precipitates.

### DNA Extraction, 16S Amplicon Sequencing, and Analysis

Genomic DNA was extracted using DNeasy® PowerWater® Kit following the manufacturer’s protocol. Bacterial diversity profiling was performed by sequencing 16S V1-V3 region amplicons on Illumina MiSeq platform using primer pairs 27 F (5′‒AGAGTTTGATCMTGGCTCAG‒3′) and 519 R (5′‒GWATTACCGCGGCKGCTG‒3′) at Australian Genome Research Facility, Melbourne, Victoria 3000, Australia. Diversity profiling analysis was performed using QIIME 2 2019.7 [31]. The demultiplexed raw reads were primer trimmed and quality filtered using the cutadapt plugin followed by denoising with DADA2 [32] (via q2‐dada2). Taxonomy was assigned to ASVs using the q2‐featureclassifier classify‐sklearn naïve Bayes taxonomy classifier [33].

The dominant ASVs were identified from ref-seq outputs. Pairwise sequence similarities were calculated according to the method recommended by Meier-Kolthoff [34] for 16S rRNA gene available via the GGDC web server [35] available at http://ggdc.dsmz.de/. Phylogenies were inferred by the GGDC web server available at http://ggdc.dsmz.de/ using the DSMZ phylogenomics pipeline [36] adapted to single genes. A multiple sequence alignment was created with MUSCLE [37]. Maximum likelihood (ML) and maximum parsimony (MP) trees were inferred from the alignment with RAxML [38] and TNT [39]. Rapid bootstrapping in conjunction with the autoMRE bootstopping criterion [40] and subsequent search was made for the best tree. For MP, 1000 bootstrapping replicates were used in conjunction with tree-bisection-and-reconnection branch swapping and ten random sequence addition replicates. The sequences were checked for a compositional bias using the *χ*^2^ test as implemented in PAUP* [41]. The phylogenetic tree was annotated and visualized using FigTree, version 1.4.4 (http://tree.bio.ed.ac.uk/).

### Naphthalene and Pyrene Biodegradation Coupled with Sulfate Reduction

Batch biodegradation experiment for periodic estimation of the residual PAH was performed in serum bottle having 15 mL effective volume with 8.75 mL mineral medium, 1.0 mL enrichment culture as inoculum, and 0.25 mL of 8.0 mM naphthalene or 5.0 mM pyrene solution supplied as HMN overlay to provide an initial concentration of 200 µM naphthalene or 125 µM pyrene. To determine the sulfate reduction activity, similar incubations in 30 mL final volume were included. Three sets of controls with (a) no inoculum (abiotic control), (b) HMN as carbon source, and (c) 20 mM Na_2_MoO_4_.2H_2_O were also maintained. The bottles were closed with rubber stoppers, secured with aluminum crimp seals, and incubated at 25 °C in inverted position. Periodically, triplicate bottles were withdrawn at 30-day intervals for reliable estimation of PAHs residues. At the same sampling interval, aliquots (2.0 mL) were withdrawn under anaerobic conditions for the estimation of sulfate and sulfide.

### Chemical Analyses

Naphthalene and pyrene were extracted by ultrasound-assisted liquid–liquid extraction procedure using hexane as the solvent. Briefly, the rubber stopper of the serum bottle was removed, and 3.0 mL hexane was added to the serum bottle, recapped and vigorously vortexed for 1.0 min. The contents were transferred to a 40 mL EPA vial. Subsequently, hexane (7 mL) was added to the serum bottle, vortexed for 30 s, and the contents were transferred to the EPA vial. The above steps were repeated by adding 10 mL hexane to the serum bottle. The total contents (30 mL:10 mL culture and 20 mL hexane) in the EPA vial were vigorously vortexed for 2.0 min and sonicated at 40 Hz for a total of 15 min in three cycles. The organic phase was separated and dried over anhydrous Na_2_SO_4_. The pyrene extract was evaporated under gentle stream of N_2_ in a temperature-controlled evaporator, and finally, the residues were dissolved in 1.0 mL hexane. Due to the high volatility, the evaporation step was skipped for naphthalene. The hexane extract was diluted and analyzed by GC–MS. One microliter sample was injected into an Agilent 7890B gas chromatograph (GC) equipped with a HP–5MS capillary column (30 m length, 0.25 mm i.d., 0.25 μm film thickness) and Agilent 7000A triple quadrupole mass spectrometer. The oven temperature was held at 40 °C for 1.0 min, then ramped to 120 °C at a rate of 25 °C min^−1^. The temperature was further increased at 10 °C min^−1^ to 200 °C and finally heated up to 300 °C at a rate of 5 °C min^−1^ and held isothermally for 10 min. Helium at a flow rate of 1.20 mL min^−1^ was used as the carrier gas. The injector temperature was 250 °C. The instrument was operated in electron impact ionization mode and monitored selective ions. No tandem mass spectrometric method was used.

Sulfate was measured spectrophotometrically according to BaCl_2_ method as described by Kolmert et al. [42] with some modifications. Briefly, the sample was diluted 1:10 with sulfate-free anoxic water. The microplate scale miniaturized reaction mixture contained 100 µL sample, 100 µL conditioning reagent [42], and 50 µL 1.0 M BaCl_2_.2H_2_O. After incubation for 1.0 min at room temperature, turbidity of BaSO_4_ was measured at 420 nm using a PerkinElmer® EnSight™ multimode plate reader. The reliability of the spectrophotometric method was evaluated by analyzing the standard sodium sulfate solutions, Dionex™ retention time standards for ion chromatography, and randomly selected samples in a Thermo Scientific™ Dionex™ Integrion™ HPIC™ system equipped with conductivity detector. Chromatographic separation was achieved in an IonPac® AS18 analytical column (4 × 250 mm) using Dionex™ EGC KOH eluent at 0.25 mL min^−1^. The variability was less than 5%; hence, the spectrophotometric method was routinely used.

Sulfide was measured spectrophotometrically in a miniaturized assay using 0.50 mL sample according to the methylene blue method [43], following the modified protocols of Reese et al. [44]. Briefly, 40 µL mixed diamine reagent was added to 500 µL sample. The reaction mixture was vortexed for 5 s, incubated at room temperature for 20 min, and diluted in water as required. Absorbance of the methylene blue complex was measured on 200 µL reaction mixture at 663 nm using a microplate reader. Due to the formation of insoluble precipitates after the addition of mixed diamine reagent, sulfide in the molybdate-amended control samples was determined according to the colloidal CuS method [45].

### Sequence Accessions

The raw sequence reads of 16S V1-V3 region amplicons, obtained using triplicate gDNA templates of MMNap and MMPyr, have been submitted to NCBI Sequence Read Archive (SRA) under the BioProject accession number PRJNA799593 with the individual accession numbers: SAMN25182043, SAMN25182044, SAMN25182045, SAMN25182046, SAMN25182047, and SAMN25182048.

## Results and Discussion

The MGP soil was neutral sandy loam contaminated with 233 mg kg^−1^ of Σ16PAHs. Almost half of the PAHs load was contributed by the four-ringed PAHs, especially pyrene (38 mg kg^−1^). Naphthalene concentration was below the detection limit. The soil was not affected by seasonal oxic-anoxic shift and was exposed to air before using it as the inoculum. In a preliminary study, aerobic PAH-degrading bacterial culture was enriched from the sample. Considering the long contamination history of the sample as well as the ability of some SRB members to thrive in aerated topsoil [46, 47], we hypothesized that the soil could harbor PAHs-degrading anaerobic bacteria. Particularly, we were interested in examining whether selective enrichment of naphthalene- and pyrene-degrading SRB could result under strict sulfate-reducing conditions. Thus, attempts were made to develop enrichments that are capable of degrading naphthalene and pyrene using MGP soil under strictly anaerobic sulfate-reducing conditions.

During the selective enrichment, the PAHs were supplied in excess as HMN overlay, which served as a reservoir and protects soil microorganisms from direct PAH exposure. Both the soil incubations showed abundant sulfate reduction, as evident by the blackening of the medium. After 90 days, sulfide concentration increased by 2.80 mM in naphthalene and 2.20 mM in pyrene amended samples. However, sulfide accumulation (2.0 mM) in samples supplemented with HMN suggested that the enrichment of SRB also utilized soil-derived organic carbon or the solvent. However, sulfate reduction in HMN amended samples began to diminish from the first subculture made in fresh medium. In contrast, the samples amended with PAHs showed slow but steady consumption of sulfate and concomitant accumulation of sulfide during the incubation period. After the second subculture, sulfate reduction activity proceeded exceptionally slowly. At the end of first 90 days of incubation, no significant change in sulfide concentration was found. Moreover, the medium color turned to light pink, suggesting oxygen intrusion through the stoppers. Probably, any soil organic matter that could serve as electron donor became diluted to extinction with serial transfers, leaving the supplied PAH as the only source of carbon and energy metabolism. Accordingly, specialized anaerobic microorganisms started slowly adapting to the selective stringent environment.

The LYE medium was formulated to check for the presence and viability of SRB in the enrichment. The medium was not compatible with the reductant sodium sulfide due to the immediate formation of FeS precipitates from the ferrous salt. However, even without any added reductant, the degassed medium was helpful enough to detect the activity of sulfate reduction within a few days of incubation. Samples inoculated into the LYE medium from the barely growing second subcultures resulted in rapid growth and FeS formation in about 2 days. Based on this circumstantial evidence, the enrichment process was continued. After 200 days, growth in both the SRB enrichments was confirmed based on the sulfide accumulation and the appearance of biomass as flakes at the HMN-PAH and medium interface. The next subcultures were made at 150 days intervals when the accumulated sulfide concentration reached ≥ 1.0 mM. In all, the selective enrichments obtained after six subcultures over a period of 2.5 years from MGP soil that could degrade naphthalene and pyrene were designated as MMNap and MMPyr, respectively.

**Fig. 1:**
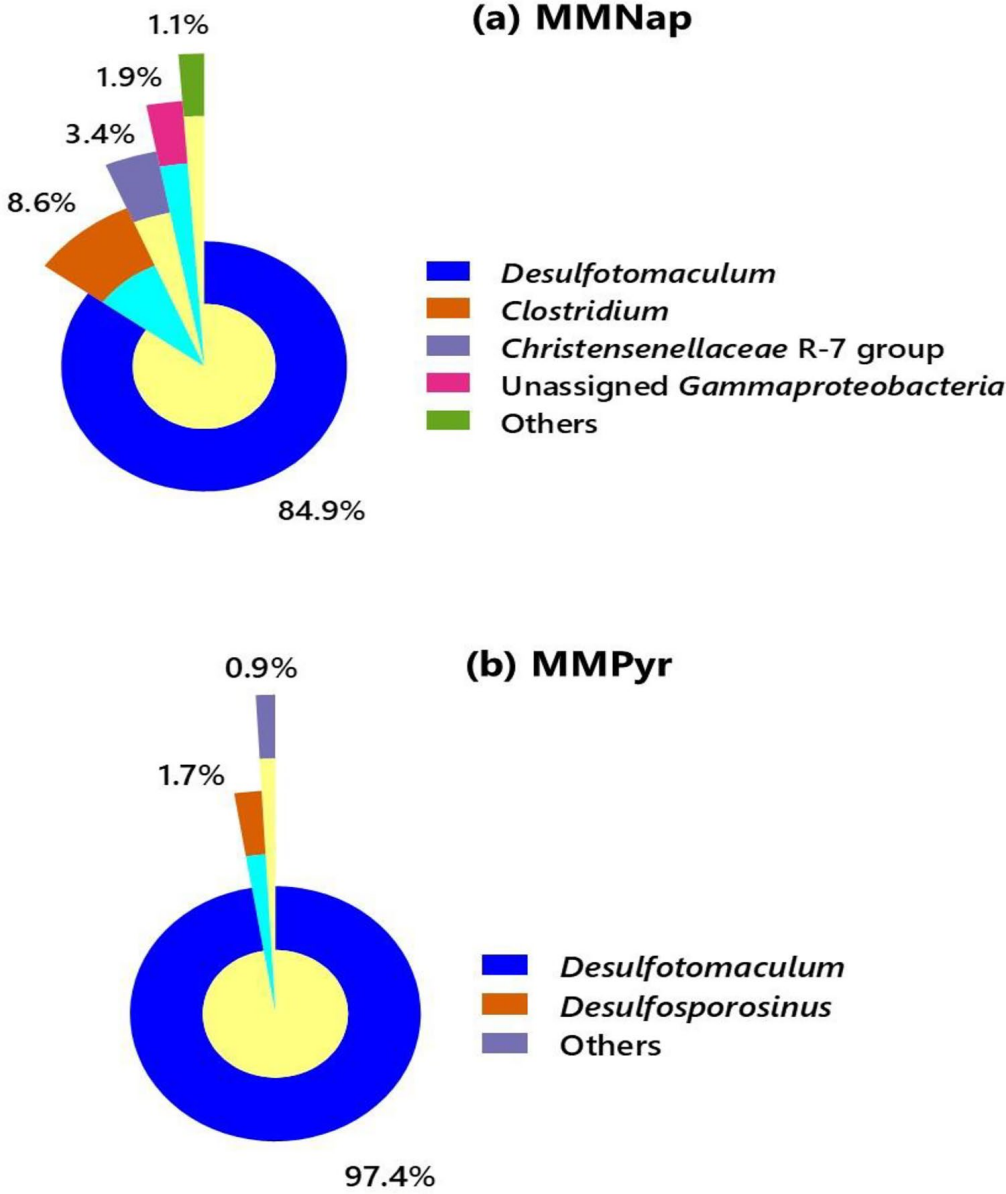
Bacterial community composition in **a** naphthalene-degrading, sulfate-reducing enrichment culture MMNap, and **b** pyrene-degrading, sulfate-reducing enrichment culture MMPyr, at the genus level. Taxonomic units counted at ≤ 1% relative abundance are combined and shown as “others” category

Bacterial community analyses were performed based on 16S V1-V3 region amplicon metagenome sequences. Triplicate samples were sequenced to avoid sequencing biases introduced from variable sequencing depth. The representative sample with adequate sequencing depth and the leveled-off plateau was identified from alpha rarefaction curves generated in QIIME 2.0 analysis. From the representative of MMNap (SRA accession number SAMN25182043), 211827 raw reads were generated, and after quality filtering, denoising, and chimera check, 166549 reads were analyzed. Sequencing the MMPyr representative (SRA accession number SAMN25182046) generated 129363 raw reads; among them, 102885 reads were analyzed after quality control processing. Bacterial diversity in MMNap and MMPyr at the genus level is shown in Fig. 1a and b, respectively.

**Fig. 2:**
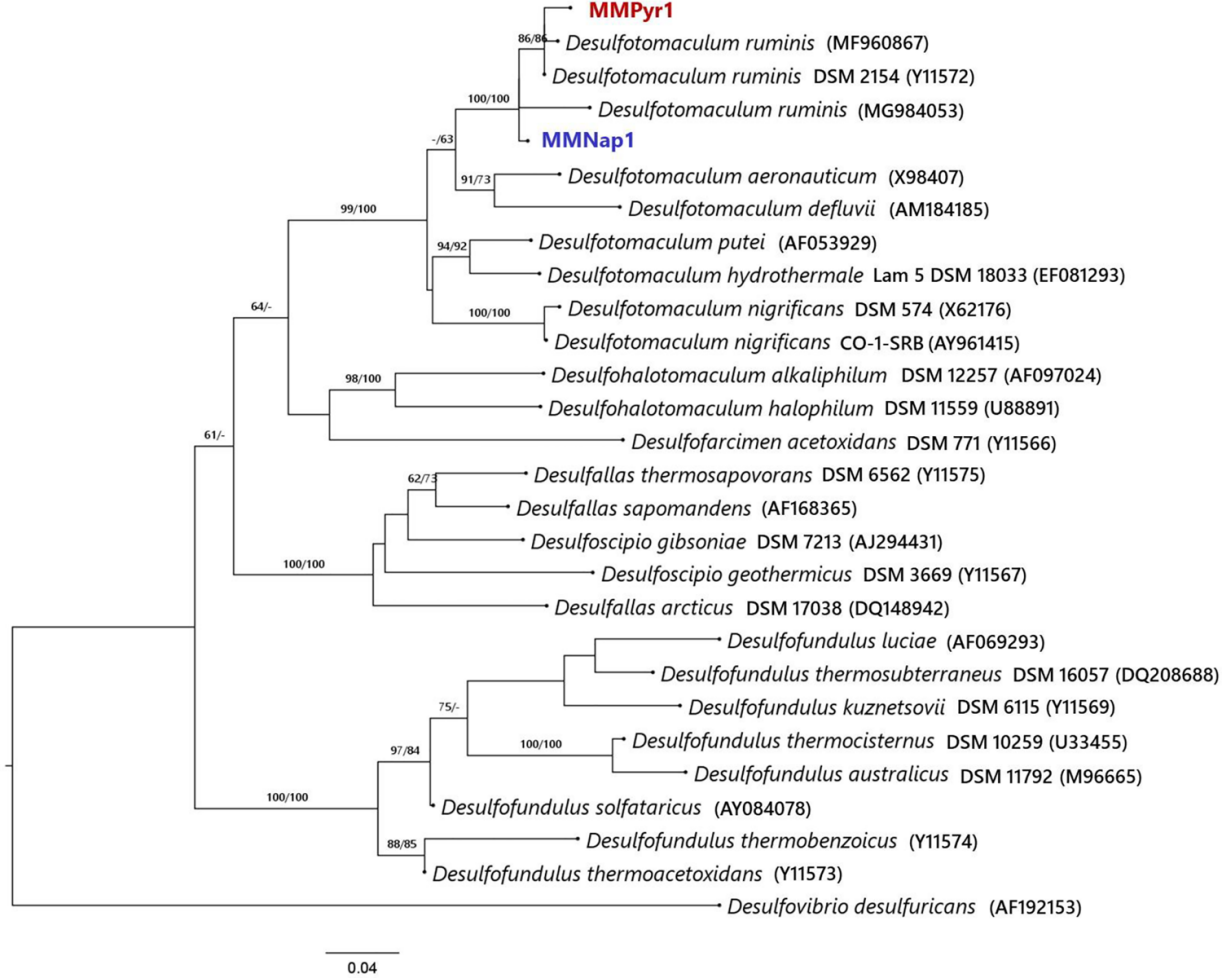
Phylogenetic positions of *Desulfotomaculum* MMNap1 and MMPyr1 among known strains of *Desulfotomaculum* in ML tree. The tree was rooted by midpoint-rooting. The branches are scaled in terms of the expected number of substitutions per site. The numbers above the branches are bootstrapping support values > 60% for ML (left) and MP (right). The input nucleotide matrix comprised 28 operational taxonomic units. The base-frequency check indicated no compositional bias (*p* = 0.88, *α* = 0.05). ML analysis under the GTR + GAMMA model yielded the highest log likelihood of − 11755.97, whereas the estimated alpha parameter was 0.28. The ML bootstrapping converged after 900 replicates; the average support was 70.36%. MP analysis yielded a best score of 2030 (consistency index 0.59, retention index 0.65) and two best trees. The MP bootstrapping average support was 70.40%

The enrichment, MMNap, was dominated by a member of the genus *Desulfotomaculum* (84.90%) (Fig. 1a). The typical soil anaerobe, *Clostridium*, accounted for less than one-tenth of the abundance, while an unassigned member of the *Chistensenellaceae* R7 group was less abundant (3.40%). The taxonomic affiliation of the Gammaproteobacterial member, which occurred at very low abundance (1.90%), could not be resolved. Although a total of twenty phylotypes, excluding the singletons and doubletons, were found at the genus level, sixteen of them combinedly constituted only 1.10% of the MMNap community (Fig. 1a). In contrast, the MMPyr community appeared to be more selectively enriched. Among the twelve phylotypes, a member of the genus, *Desulfotomaculum*, was found to be predominant (97.40%) (Fig. 2b), while *Desulfosporosinus* constituted only a minor (1.70%) of the community.

The sequences of *Desulfotomaculum* sp*.* in both the enrichments, designated as MMNap1 and MMPyr1, showed 98.80% sequence similarity to each other. This observation suggests that they could be the same strain of the genus. Phylogenetic positions of the MMNap1 and MMPyr1 among known strains of *Desulfotomaculum* is shown in Fig. 2. Both were found to be related to the strains of *D. ruminis.* However, MMNap1 shared only 96.56% similarity with *D. ruminis* RZ12 (MG984053), while MMPyr1 showed only 95.88% similarity with *D. ruminis* X44 (MF960867). The sequence similarity of MMNap1 and MMPyr1 with the type strain, *D. ruminis* DSM 2154 (Y11572), was 96.22 and 95.80%, respectively. Since the sequence similarity scores and phylogenetic positions were determined from partial 16S rRNA sequences (MMNap1 and MMPyr1, both 408 nucleotides), analysis of full-length sequences would be necessary to infer accurate phylogenies.

The extent of naphthalene and pyrene degradation and concomitant changes in sulfate and sulfide were measured in batch experiments. During the enrichment phase, both the PAHs were provided in excess concentrations. Generally, PAH residues are estimated by withdrawing few microliters from HMN-PAH droplet and directly injecting them into the instrument [16]. However, repeated injections of such a highly concentrated sample may contaminate the column that, in turn, may severely impact the detector, especially the mass spectrometer. In this study, PAHs were extracted from the whole incubation, diluted appropriately to match the instrument detection, and injected into GC–MS system that allowed reliable estimation avoiding potential overestimation from carryover. The initial concentration (200 µM naphthalene, 125 µM pyrene) was selected based on the observed residual amounts of PAHs and sulfide accumulation in the sixth enrichment.

**Fig. 3:**
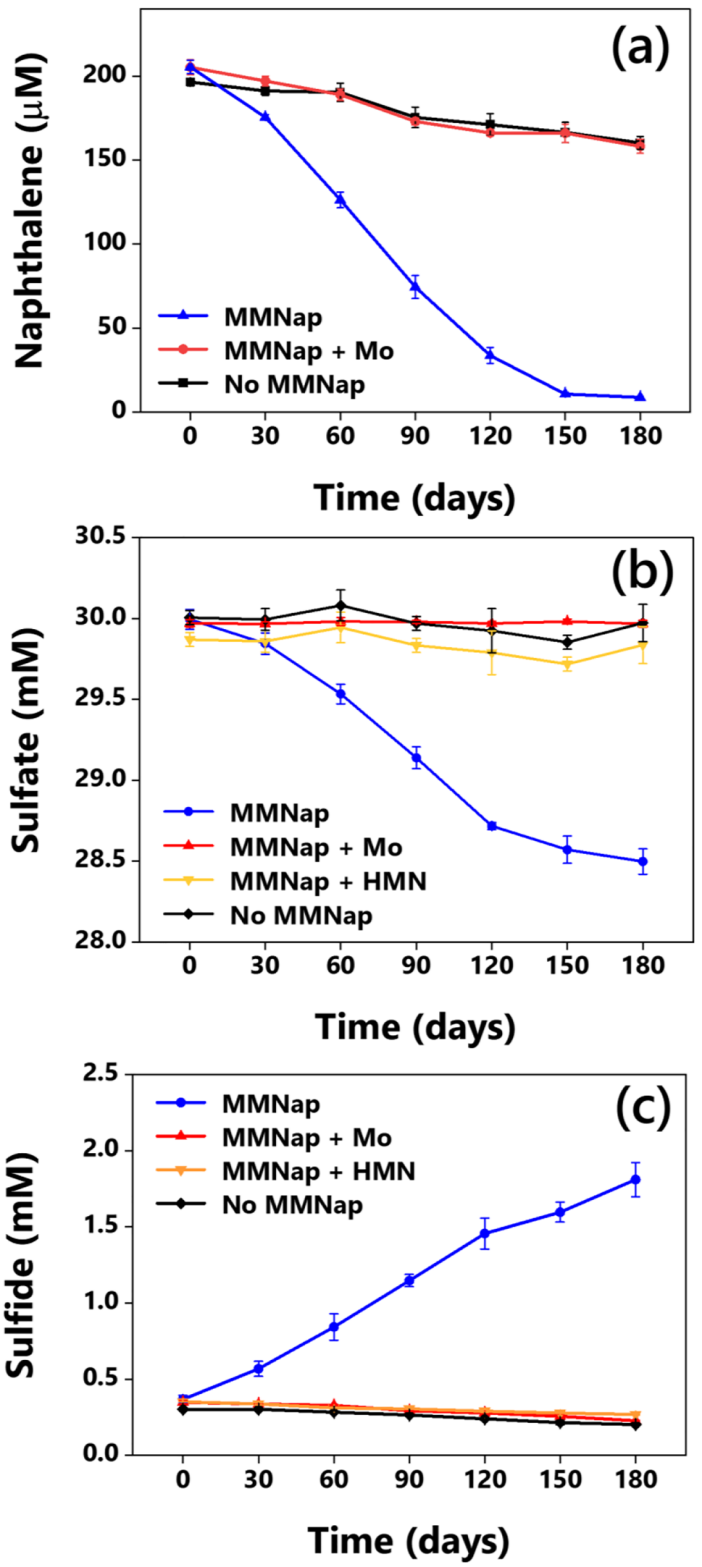
Anaerobic naphthalene degradation by MMNap measured in terms of **a** naphthalene consumption, **b** sulfate consumption, and **c** sulfide accumulation. Data represent mean ± standard deviation obtained from three independent replicates

Naphthalene degradation from the sample inoculated with MMNap started without any apparent lag and proceeded slowly. After 150 days, almost 195 µM naphthalene was degraded by the enrichment (Fig. 3a). To ascertain the involvement of sulfate reducer in naphthalene biodegradation, molybdate (Mo) at 20 mM concentration was added to a separate set to inhibit the bacterial sulfate reduction [48]. No significant degradation of naphthalene was observed in inoculated samples amended with 20 mM Mo when compared with the samples inoculated with the enrichment in culture medium with no added Mo (Fig. 1a). The apparent depletion of naphthalene from abiotic and Mo-amended samples is probably due to the loss of this volatile compound during the extraction process and adsorption to the stoppers during prolonged incubation. Naphthalene utilization by MMNap was coupled to consumption of sulfate (Fig. 3b) and accumulation of sulfide (Fig. 3c) during incubation. Lack of sulfate reduction activity in HMN-amended control samples suggest that the solvent did not serve as an electron donor or carbon source. Naphthalene degradation by the sulfate-reducing MMNap could also be inferred based on the absence of sulfate reduction in Mo-amended control (Fig. 3b and c).

**Fig. 4:**
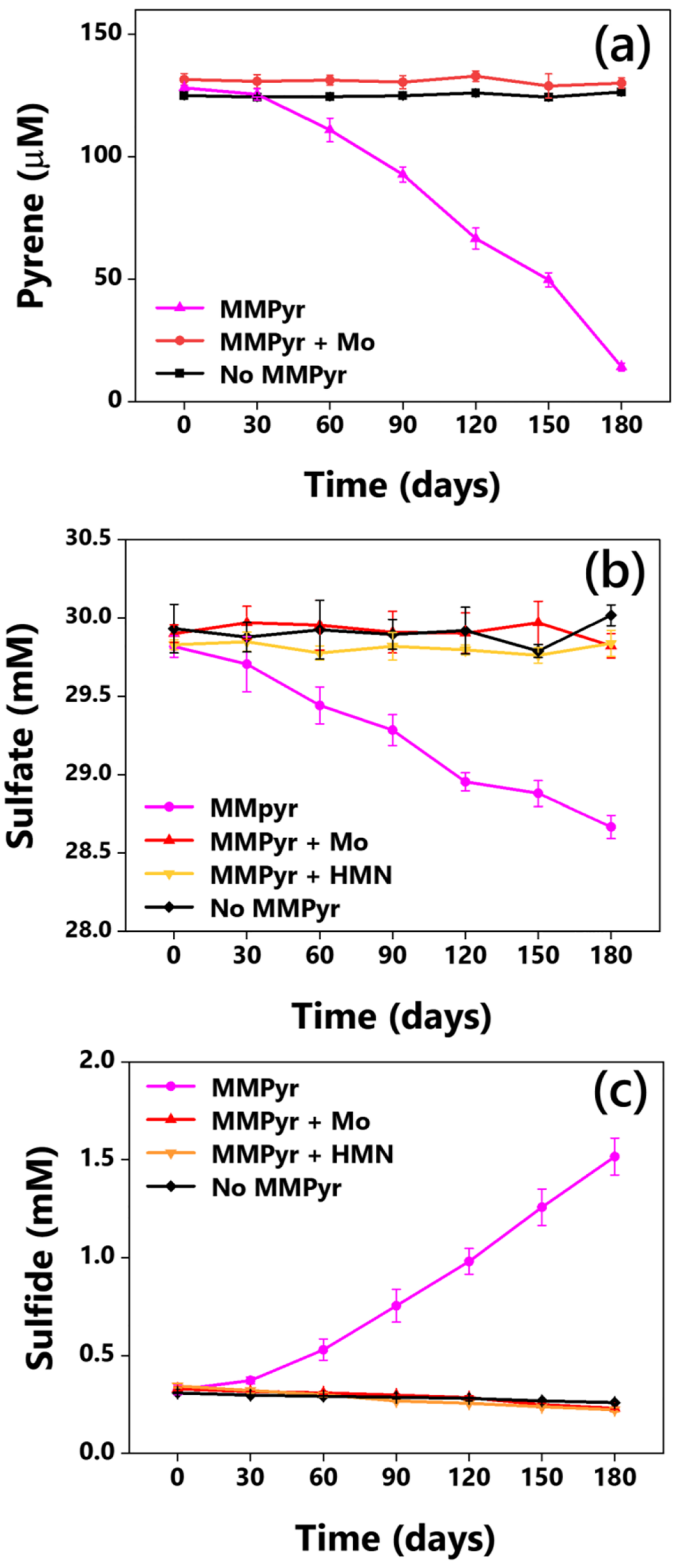
Anaerobic pyrene degradation by MMPyr determined in terms of **a** pyrene consumption, **b** sulfate consumption, and **c** sulfide accumulation. Data represent mean ± standard deviation obtained from three independent replicates

MMPyr degraded almost 114 µM of the substrate pyrene during 180 days of incubation. An initial lag of about 30 days was observed, during which only 3.0 µM pyrene was removed. However, during the next 150 days, pyrene was gradually removed at approximately the same rate. Pyrene concentration remained constant in both Mo-amended and abiotic controls, which indicates the central role of sulfate reducers in the pyrene removal (Fig. 4a). Towards the end of the incubation, 1.04 mM sulfate was depleted from the medium (Fig. 4b) with a concomitant increase in sulfide concentration by 1.19 mM (Fig. 4c). In both the cases, substrate depletion was associated with sulfate reduction and is in close agreement with the theoretical estimation derived from the reaction stoichiometry (Table 1). During 180 days of incubation, 1 mol of naphthalene degradation by MMNap required 9.30 mol of sulfate and produced 8.97 mol sulfide. In the case of pyrene degradation by MMPyr, 1 mol of electron donor utilization resulted in the depletion of 10 mol of electron acceptor for the yield of 10.45 mol sulfide. It should be noted that the residues were estimated from 10 mL cultures, including 1.0 mL (10%) of inoculum, whereas sulfate and sulfide were estimated from the samples prepared in 30 mL cultures, including 3.0 mL (10%) inoculum. Thus, the apparent higher ratio of substrate degradation to sulfate depletion or sulfide accumulation is probably due to the differences in initial PAHs that resulted from variations in HMN-PAH carryover along with the inocula.

**Table 1:** Stoichiometry of anaerobic degradation of naphthalene and pyrene coupled with sulfate reduction

Parameter	Unit
*(a) Anaerobic naphthalene degradation under sulfate-reducing conditions* C_10_H_8_ + 6SO_4_^2−^ + 6H_2_O → 10HCO_3_^−^ + 6HS^−^ + 4H^+^	
Naphthalene net consumed (µmol)	160.70
Sulfate net consumed (µmol)	1495.80
Naphthalene net consumed: sulfate net consumed	1:9.30
Sulfide accumulated (µmol)	1441.60
Naphthalene net consumed: sulfide net accumulated	1:8.97
*(b) Anaerobic pyrene degradation under sulfate-reducing conditions* C_16_H_10_ + 9.25SO_4_^2−^ + 11H_2_O → 16HCO_3_^−^ + 9.25HS^−^ + 6.75 H^+^	
Pyrene net consumed (µmol)	114.00
Sulfate net consumed (µmol)	1149.60
Pyrene net consumed: sulfate net consumed	1:10
Sulfide net accumulated (µmol)	1191.40
Pyrene net consumed: sulfide net accumulated	1:10.45

*Desulfotomaculum* spp*.* are metabolically versatile, endospore-forming, and anaerobic SRB [13, 26]. Taxonomically, they are affiliated with the phylum *Firmicutes*, class *Clostridia*, order *Clostridiales*, and family *Peptococcaceae* [49]. Currently, the genus contains 36 validly published child taxa, including members adapted to extreme temperature, salinity, and pH. *Desulfotomaculum* spp. have been reported in diverse habitats involving culture-dependent and culture-independent studies [13]. They are notable for their ability to survive and colonize in inhospitable environments such as deep subsurface and oxic topsoil, where sulfate concentration is generally low [13, 26]. In habitats under the influence of seasonal variations in oxygen, *Desulfotomaculum* spp*.* flourish upon the return of anoxic conditions [26]. Thus, when the contaminated MGP soil was incubated under strictly anaerobic sulfate-reducing conditions with naphthalene or pyrene as a carbon source, the PAHs-degrading sulfate-reducer *Desulfotomaculum* MMNap1 and MMPyr1 were enriched. The *Clostridium* phylotype in MMNap enrichment is also a Gram-positive soil bacterium but rarely reported as a sulfate reducer [50]. It should be noted that certain *Desulfotomaculum* spp*.* can also grow autotrophically with H_2_/CO_2_ and sulfate [13]. However, none of the enrichments described in this study could grow and reduce sulfate on H_2_/CO_2_ present in the anaerobic workstation atmosphere (N_2_:CO_2_:H_2_, 80:10:10) under the experimental conditions.

The involvement of the members of *Desulfotomaculum* spp*.* in the anaerobic biodegradation of hydrocarbons has been reported in a few studies. *D. gibsoniae* strain Groll isolated from freshwater ditch could degrade phenol, *p*-cresol, 4-methylcatechol, and catechol [51–53]. Based on dissimilatory sulfate reductase gene (*dsrAB*) profiling, Pérez-Jiménez et al. [54] proposed the participation of phylotypes related to *Desulfotomaculum* spp*.* in naphthalene, 2-methylnaphthalene, and phenanthrene-degrading sulfate-reducing enrichment cultures. In another instance, the role of *Desulfotomaculum* phylotype present in an ethylcyclopentane degrading enrichment was proposed [55]. Recently, phenanthrene-degrading sulfate-reducing pure culture of *Desulfotomaculum* sp. PheS1 has been reported [19]. To date, anaerobic pyrene degradation has been reported in sediment microcosms [21, 22] and sludge microcosms [20, 24]. However, the microcosms were highly diverse in microbial compositions. Four strains of *Clostridium* spp*.* were claimed as capable of pyrene degradation under anerobic conditions; but no direct relationship between substrate degradation and sulfate reduction was evident [23]. Thus, the current study is the first report on pyrene degradation by any soil-free, strictly anaerobic sulfate-reducing enrichment culture. Future investigation will be directed toward mechanistic understanding of anaerobic degradation processes of PAHs.
